# Neurofilament light chains to assess sepsis-associated encephalopathy: Are we on the track toward clinical implementation?

**DOI:** 10.1186/s13054-023-04497-4

**Published:** 2023-05-31

**Authors:** Barbora Bircak-Kuchtova, Ha-Yeun Chung, Jonathan Wickel, Johannes Ehler, Christian Geis

**Affiliations:** 1 Section Translational Neuroimmunology, Department for Neurology, Jena University Hospital, Am Klinikum 1, 07747 Jena, Germany; 2 Center for Sepsis Control and Care, Jena University Hospital, 07747 Jena, Germany; 3 Department of Anesthesiology and Intensive Care Medicine, Jena University Hospital, 07747 Jena, Germany

**Keywords:** Sepsis, Delirium, Neurofilament light chain, Biomarker, Encephalopathy, Brain dysfunction, SAE

## Abstract

Sepsis is the most common cause of admission to intensive care units worldwide. Sepsis patients frequently suffer from sepsis-associated encephalopathy (SAE) reflecting acute brain dysfunction. SAE may result in increased mortality, extended length of hospital stay, and long-term cognitive dysfunction. The diagnosis of SAE is based on clinical assessments, but a valid biomarker to identify and confirm SAE and to assess SAE severity is missing. Several blood-based biomarkers indicating neuronal injury have been evaluated in sepsis and their potential role as early diagnosis and prognostic markers has been studied. Among those, the neuroaxonal injury marker neurofilament light chain (NfL) was identified to potentially serve as a prognostic biomarker for SAE and to predict long-term cognitive impairment. In this review, we summarize the current knowledge of biomarkers, especially NfL, in SAE and discuss a possible future clinical application considering existing limitations.

## Background—Sepsis associated encephalopathy

Sepsis is a potentially life-threatening organ dysfunction caused by a dysregulated host response to severe infection [1, 2]. Due to disease severity, delayed diagnosis and limited effective therapeutic strategies, sepsis is still associated with high mortality rates up to 30% and even up to 50% in patients with septic shock [2, 3]. According to the analysis for the Global Burden of Disease Study from 2017, sepsis was estimated to be associated with almost 20% of global deaths [4]. Over the past years, substantial preclinical and clinical research contributed to increased awareness and optimized therapeutic standards especially in intensive care units (ICUs) to improve the outcome of sepsis patients [2]. However, patients surviving critical illness after long-term ICU treatment, and in particular sepsis survivors, often suffer from long-term sequelae. The syndrome of post-intensive care (PICS) includes a heterogeneous symptom complex consisting of neuromuscular weakness, mental health issues (e.g., post-traumatic stress disorder, anxiety, depression) and neurocognitive dysfunction [5–9]. Moreover, intensive care survivors may also experience further symptoms, such as osteopenia, dysphagia, fatigue, pain, and metabolic disorders. Therefore, it was suggested to extend the definition of PICS [10]. Sepsis survivors suffer from similar symptoms while long-term cognitive dysfunction has been shown to be a major and frequent complication [11, 12]. These long-term sequelae not only affect patients and their primary caregivers, but also create a considerable socioeconomic burden for the public health care system [13–15].

Sepsis-associated encephalopathy (SAE) is one of the most common organ dysfunctions in sepsis and is also associated with significantly higher mortality rates [12, 16–18]. The term SAE is derived from human post-mortem studies and animal models. It includes functional brain deficits and neuronal injury during the course of systemic inflammation [11]. From a clinical perspective, delirium is the most common SAE syndrome and it is characterized by acute and diffuse brain dysfunction with changes in attention, disorientation, halluzination, agitation, or even coma [11, 18–20]. Since delirium can have multifactorial etiologies other than sepsis, SAE is a diagnosis of exclusion [19, 20]. Thus, differential diagnoses, such as direct infection of the central nervous system, trauma, non-convulsive status epilepticus, or drug side effects need to be ruled out using also apparative diagnostics, e.g., electroencephalography, brain imaging (computertomography or magnetic resonance imaging), detailed laboratory tests and, if necessary, cerebrospinal fluid (CSF) analyses [11, 21, 22].

Due to inconsistent diagnostic criteria in clinical studies and diverging daily clinical practice, the estimated prevalence of delirium in SAE varies from 9 to 71% in sepsis patients [16]. As an example, a multicenter study including 2513 patients identified cerebral dysfunction due to SAE in approximately 50% of sepsis patients on the ICU [18]. In contrast, a large prospective sepsis cohort with 3210 patients diagnosed delirium in approximately 33% using the Confusion Assessment Method for the Intensive Care Unit (CAM-ICU) and Nursing Delirium Screening Scale (Nu-DESC) as the most reliable clinical scores [23].

In addition to acute changes in mental state during the acute phase, sepsis is associated with long-term cognitive dysfunction following hospital discharge. According to a study of long-term cognitive outcomes in patients admitted to ICUs with acute respiratory failure, septic or cardiogenic shock, 40% (after three months) and 34% (after twelve months) of patients had persisting cognitive impairment after hospital discharge [12]. The severity of cognitive impairment was worse than typically seen in patients with moderate traumatic brain injury, and in 26% (after three months) and 24% (after twelve months) of patients, the cognitive impairment was comparable to mild Alzheimer’s disease [12, 24]. Similarly, an Australian prospective cohort study found approximately 40% of participants cognitively impaired three months after ICU-discharge with improvement to 20% at six-months after ICU-discharge in ICU patients. The slightly lower rate of patients with persistent cognitive dysfunction might be due to lower incidence of ICU delirium in this study with only 19%, which is most likely caused by different characteristics of the patient population (e.g., comorbidities, age and lower severity of illness) [25]. The duration and severity of delirium due to SAE is a known predictor for development of long-term cognitive impairment [11, 12, 26, 27]. Over 70% of patients with delirium and ICU stay still suffered from cognitive impairment after one year, with delirium duration being an independent predictor of worse cognitive performance [26]. The risk of acquiring moderate to severe cognitive impairment was found to be 3.3 times higher following an episode of sepsis, with additional increase in those patients with preexistent cognitive dysfunction [5]. SAE and long-term brain dysfunction also frequently cause higher level of care dependency in elderly patients and affect the activities of daily living [24]. Besides impaired cognitive function, preexisting psychiatric disorders, such as depressive, anxiety and trauma-and-stressor-related disorders, are also associated with a prolonged ICU stay in the context of sepsis [28, 29].

### Challenges in the SAE assessment using clinical scores in the ICU

In the ICU, delirium severity is routinely measured using the CAM-ICU and Intensive Care Delirium Screening Checklist (ICDSC), both showing high reliability and validity in patients who are able to interact with the investigator. According to CAM-ICU, a patient is rated as delirious when (a) the mental status acutely changes or fluctuates and (b) the patient fails to pay attention and either (c) the patient exhibits disorganized thinking or (d) has an altered level of consciousness. To evaluate the level of consciousness, the Richmond Agitation-Sedation Scale (RASS) is used [30]. Another routinely used scoring tool, the ICDSC, is an eight-item checklist based on criteria of the Diagnostic and statistical manual of mental disorders (DSM) and features of delirium: altered level of consciousness, inattention, disorientation, hallucination or delusion, psychomotoric agitation or retardation, inappropriate mood or speech, sleep/wake cycle disturbance, and symptom fluctuation [31]. However, when compared to CAM-ICU, the ICDSC has “only” moderate sensitivity and good specificity (sensitivity and specificity of 80% and 95.9% vs. 74% and 81.9%, respectively) [32]. Another delirium assessment instrument is the Nu-DESC, which is based on the observation of patients and consists of a 5-item scale derived from Confusion Rating Scale (CRS). The CRS is a brief nursing delirium screening test rating symptoms such as disorientation, inappropriate behavior and communication as well as hallucinations. The initial version of the CRS showed good results in delirium screening, however, it neglected hypoactive patients. Therefore, a fifth item scoring psychomotor retardation was added [33].

The major disadvantage of the CAM-ICU and the other scores is the reliance on patient interaction. Therefore, these clinical tools cannot be used in deeply sedated patients (e.g., patients with RASS of -4 and -5 are ineligible for CAM-ICU). Furthermore, patients with hypoactive delirium might also be underrecognized. Thus, the delirium rates in the ICU are likely to be underestimated in severely ill patients.

Besides challenges in the practical use of delirium tests itself in SAE patients, a recent study evaluated the current practice of clinical SAE diagnostics in the ICU and demonstrated a great heterogeneity in the application of diagnostic tests in Germany [34].

### Neuronal damage and impaired cognitive function in SAE

**Fig. 1:**
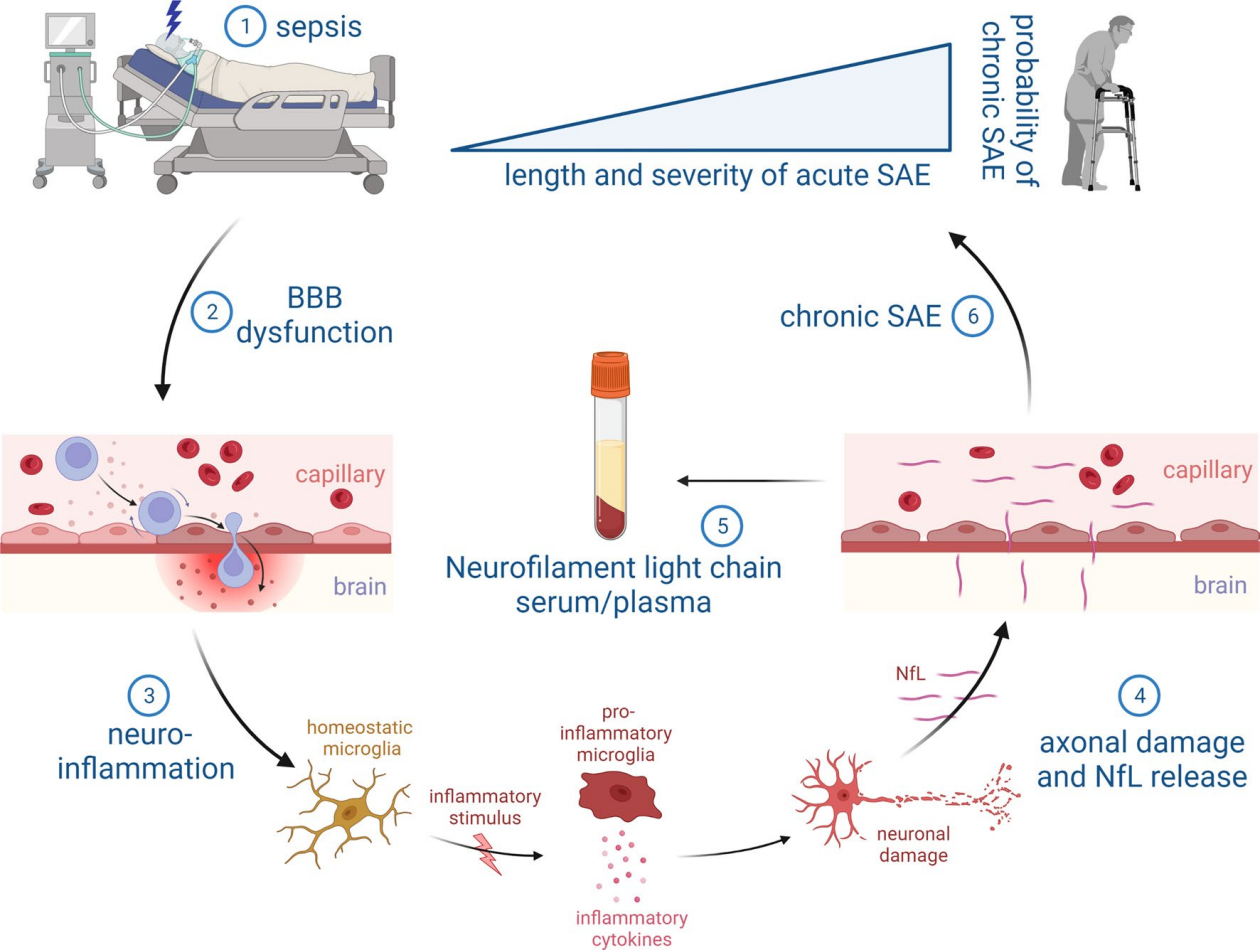
Underlying pathophysiology of NfL increase in serum/plasma during the course of sepsis and its possible implication in the diagnosis and severity assessment of SAE

Both human autopsy and *in-vivo* data indicate neuronal and synaptic damage in sepsis alongside with activation of immune cells in the CNS. Analyses of brain autopsies from patients who died of sepsis showed diffuse cerebral damage with neuronal apoptosis, axonal damage and ischemic lesions [35–38]. Additionally, proliferation and activation of glial cells, such as microglia and astrocytes, is evident in brain tissue of sepsis patients (Fig. 1) [39, 40]. Moreover, a recent study analyzing CSF in patients with infectious delirium and Alzheimer’s disease showed an overlap in protein expression patterns in delirium and Alzheimer’s disease patients. This included a downregulation of synapse-associated protein expression and a loss of homeostatic microglia control suggesting an overlapping pathophysiology [41]. It was suggested that the evident neuronal and synaptic damage during sepsis results in memory impairment and neurocognitive dysfunction in sepsis survivors. Such impairments include deficits in spatial memory [42], impairment of verbal learning and memory [43, 44], executive functions [43, 45], pattern recognition memory, delayed-matching-to-sample tests [42], attention, and vigilance [43]. These neurocognitive deficits may be long-lasting and even irreversible, thus significantly affecting daily life of sepsis survivors and their primary caregivers [5, 42].

Interestingly, these findings in humans are corroborated in murine sepsis models and mechanistic analyses showed disordered neuronal transmission and brain network function together with neuronal damage, loss of dendritic spines and synapses as well as microglia activation [46–53]. As a consequence, specific learning and memory tasks, e.g., object recognition and spatial memory, are impaired in post-septic mice [49, 53, 54].

Still, the upstream processes that ultimately lead to synaptic and neuronal damage are only insufficiently understood in humans and mice. A combined pathology including disruption of the blood brain barrier, hemorrhagic/ischemic lesions, impaired neurotransmission, and neuroinflammation likely contribute to the development of neuronal injury finally causing brain and cognitive dysfunction (Fig. 1) [21].

### Biomarkers of SAE

Biomarkers should provide objective and quantitative results. In SAE, an ideal biomarker would provide high sensitivity and specificity independent of the effects of sedatives. It should enable an early diagnosis as well as a reliable outcome assessment. Several potential biomarkers have been proposed and studied in the course of SAE, such as neuron- and glia-derived proteins [55, 56], but also inflammation-associated biomarkers [57]. These candidates were investigated in order to improve early detection of delirium and encephalopathy [58]. In addition, there have also been attempts to predict long-term cognitive outcomes in critically ill patients using a panel of inflammation- and coagulation-associated biomarkers. However, these have largely failed to provide sufficient evidence [59]. Importantly, biomarkers should be detectable in the blood instead of CSF allowing easy and repetitive measurements and quantitatively reflect brain damage. Among those candidates, S-100ß—an astrocytic marker protein indicating blood brain barrier disruption and neuronal injury—was found to be increased in SAE patients and associated with higher mortality in some studies [57, 60–63], while others showed no correlation between S-100ß increase and SAE severity [64, 65]. Since S-100ß is predominantly synthesized by astrocytes, it should not be regarded as biomarker of direct neuronal injury and may therefore lack specificity [60]. As direct indicators of neuronal injury, neuron-specific enolase (NSE) and Tau were elevated in SAE patients [58, 63, 66]. Increased NSE levels have also been reported to be associated with delirium and poor outcome in sepsis patients [55]. Other biomarkers investigated in SAE are the astrocytic intermediate filament glial fibrillary acidic protein (GFAP), found in astrocytes and Schwann cells, and the enzyme ubiquitin carboxy-terminal hydrolase-L1 (UCH-L1), which is localized almost exclusively in neurons and neuroendocrine cells [67, 68]. GFAP and UCH-L1 in ICU patients with sepsis were associated with disease severity and predicted worse outcomes [68]. In patients with COVID-19 infection, higher plasma levels of GFAP correlated with delirium severity [69]. However, the diagnostic accuracy of these biomarkers still remained low. This might be due to the reason that S100ß protein and GFAP represent glia cell involvement and damage, but do not directly reflect neuronal injury [68, 70]. The sensitivity and/or specificity of NSE, Tau and UCH-L1 showed only insufficient prognostic results in the course of SAE [66, 68]. Studies using inflammatory markers to assess delirium in critically ill patients showed diverging results. While Ritter et al. found no association of inflammatory markers with SAE [71], Khan et al. showed that IL-6, 8, 10 were associated with the severity and duration of delirium in critically ill patients [57].

In the last decade, neurofilament light chain (NfL) has been intensively investigated as a biomarker in several neurological diseases [72]. NfL is a specific axonal injury marker and correlates well with neurodegeneration and associated symptoms, e.g., changes in cognition [73]. Therefore, NfL has been attributed to become a more appropriate biomarker for SAE with higher diagnostic accuracy [68].

## Neurofilament and neurofilament light chain

Neurofilaments are cylindrical proteins found in the neuronal cytoplasm [74]. Together with microtubules and actin filaments, neurofilaments form the neuronal cytoskeleton. Although they are also present in perikarya and dendrites, a particularly high expression of neurofilaments is found in axons where they are essential for the radial growth during the development. Their function is to provide structural support to axons. Via regulation of the axonal diameter (caliber) they also determine the conduction velocity in myelinated nerve fibers [75, 76]. Neurofilaments are composed of three subunits: NfL (low weight), NfM (medium weight) and NfH (high weight), according to their molecular mass [77–79]. In normal conditions, low levels of neurofilaments (and therefore NfL) are continuously released from the axons with association to age [80]. Neuroaxonal damage, independent of its cause, results in an increase of the neurofilament levels not only in CSF but, through the blood–brain barrier and CSF drainage into the venous system, also in the blood (serum or plasma) facilitating measurements to monitor CNS diseases [81]. The assessment of serum NfL allows the quantification of the severity of neuronal damage. Compared to invasive CSF acquisition via lumbar puncture, blood samples are easy to collect. Moreover, using the highly sensitive single molecule array (Simoa) technology a direct and high linear correlation of CSF and serum NfL values has been demonstrated [82, 83]. In the last years, series of studies investigated the value of NfL as a biomarker of neuronal injury in several neurological and non-neurological diseases [84]. In particular in inflammatory, neurodegenerative, traumatic, and cerebrovascular neurological diseases, NfL levels increase in CSF and blood proportionally to the degree of axonal damage [72]. Thus NfL has become an already established biomarker for neuronal injury and as a surrogate parameter for disease activity in neurodegenerative diseases, such as ALS, Parkinson's disease, and in multiple sclerosis. Moreover, NfL has also been used for the evaluation of disease severity and worse outcome in ischemic stroke [81, 85] and increased serum NfL levels were also found in patients with antibody-mediated encephalitis [86].

## Neurofilament light chain in SAE

Due to its proven role as a biomarker directly reflecting neuronal damage with the opportunity of blood measurements, NfL has been considered an ideal candidate for the diagnosis and prognostic assessment in SAE [71]. However, at this point, only few studies evaluated NfL in SAE and systemic inflammation. ICU patients with COVID-19 infection fulfilling the sepsis criteria showed elevated blood NfL levels, which were associated with unfavorable outcome and death [87–92]. Furthermore, a German exploratory prospective longitudinal study at three ICUs compared NfL and NfH levels in CSF and plasma of SAE patients and control patients. Whereas the values on the first day after ICU-admission showed no differences between sepsis and control patients, the levels of NfL and NfH increased significantly in the SAE group from the first to seventh day of the ICU-stay and correlated with the clinical symptoms of SAE (Fig. 1). Increased NfL levels also correlated with MRI abnormalities and survival rates. These findings have now to be confirmed in a larger prospective study because of the relatively small sample size of 20 patients with sepsis and five control patients [35, 93]. Similarly, another small prospective study also documented elevated NfL levels in 11 patients with SAE which showed mild cognitive impairment after discharge [94].

Very recently, in a gender and age-matched series of patients with community-acquired pneumonia we demonstrated that serum NfL levels were associated with the occurrence of SAE as determined by confusion or delirium but not with overall disease severity, thus supporting the specificity of Nfl as a marker for CNS involvement in infectious and inflammatory disease [95]. These findings are further supported by two other studies showing that increased NfL levels are associated to severity and length of delirium in sepsis and critically ill patients [96, 97].

Considering these results, blood NfL levels could serve as a biomarker for SAE and may have potential to predict long-term cognitive impairment after sepsis. In addition to the evidence provided by the above-mentioned first studies with rather small sample size there are several arguments that NfL might have a larger potential as compared to other biomarkers. It has already proven to be directly associated with long-term cognitive impairment and even predicting worsening of cognition over time in other neurological diseases, such as multiple sclerosis, cardiac surgery, Alzheimers’ disease and mild cognitive impairment [98–101]. Moreover, current high-sensitive technologies to determine NfL serum levels by single molecule arrays clearly improve the sensitivity and reliability of biomarker detection and evaluation in the context of neurological disease in comparison to previous attempts [74, 102].

Together, an increase of NfL levels at a defined time point during sepsis or changes in serum NfL levels over time during SAE might be suitable and clinically useful to predict long-term cognitive outcome in patients with sepsis. An NfL increase might enable ICU clinicians to identify patients at high risk for structural brain damage and therefore to prioritize brain imaging and protection during the hospital stay [34, 56]. These hypotheses now need to be tested in prospective controlled studies.

## Limitations of the use of neurofilament light chains

**Fig. 2:**
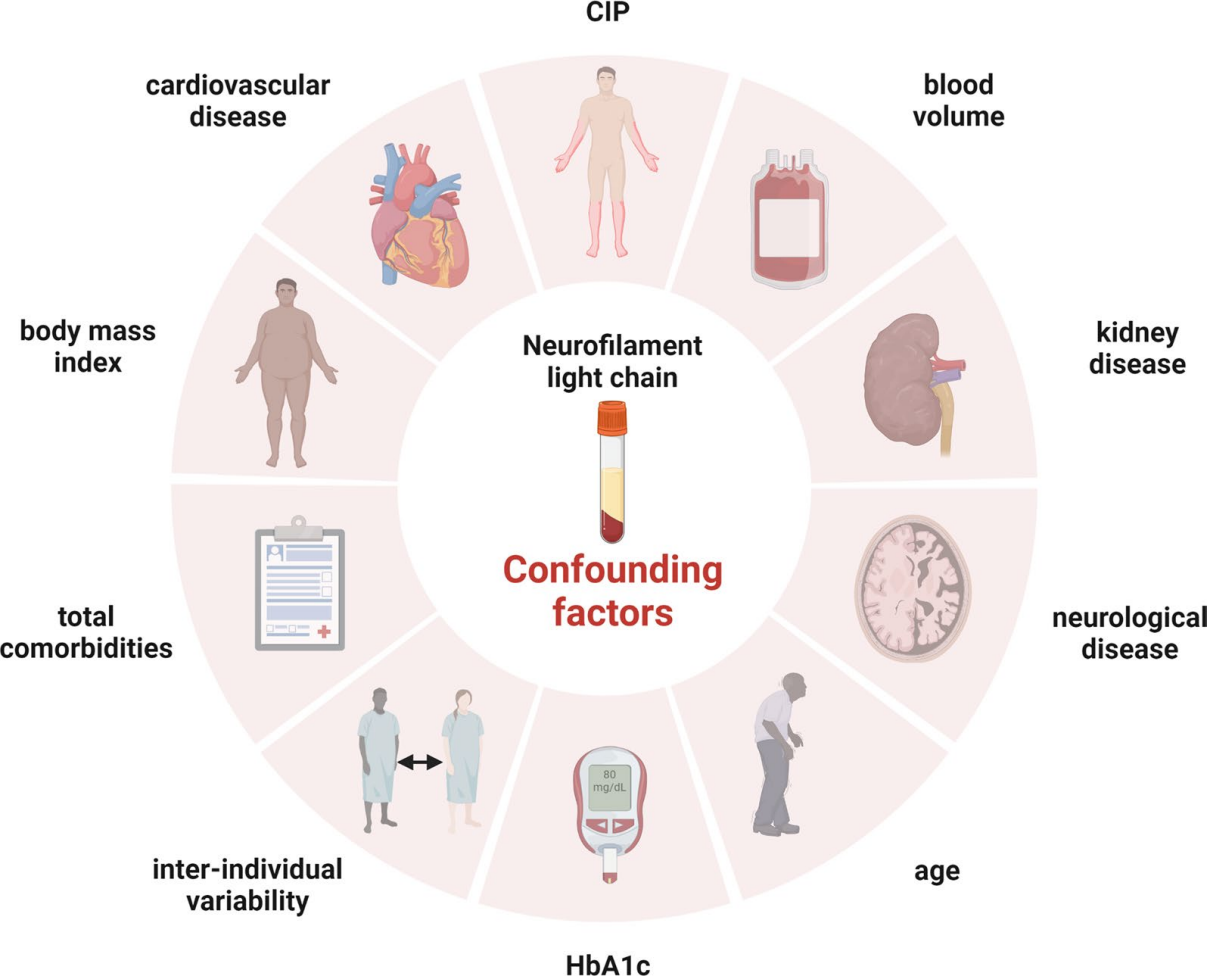
Validated confounders influencing NfL levels with potential relevance in the course of systemic inflammation

There are several factors that may influence serum levels of NfL in the setting of severely ill sepsis patients (Fig. 2). Here, NfL as a marker of neuronal injury can also be increased by ICU–acquired weakness (ICU-AW) due to peripheral nerve damage. ICU-AW is a frequent neuromuscular complication of critical illness caused by CIM, CIP or critical illness neuromyopathy (CINM). It has been shown that neurofilament levels are increased in patients with ICU-AW [103], which might affect the specificity and thus the applicability as a biomarker for SAE. Moreover, frequent neurological comorbidities in those severely ill patients with acute or chronic neuroaxonal injury in the central or peripheral nervous system will highly influence blood NfL levels [73, 104]. Even more difficult is to differentiate NfL increase due to subclinical or not yet diagnosed neurodegenerative diseases (e.g., Alzheimer’s disease, Parkinson’s disease) [105].

In general, NfL values show also interindividual variability in healthy individuals and in patients with certain comorbidities [80, 106]. The most important influential factors are age followed by renal and liver function (eGFR, urea, GPT), cardiovascular risk factors (systolic blood pressure, HDL and HbA1c), BMI and blood volume as well as total comorbidity burden (e.g., COPD, any cardiovascular disease, diabetes, kidney disease; see also Fig. 2) [107, 108]. The levels of serum NfL increase gradually with age, reduced eGFR and cerebrovascular risk factors [107]. BMI was evaluated as an important factor for decreased NfL values especially in individuals below 60 years of age, whereas an inverse relationship between serum NfL and BMI was observed particularly in underweight participants (BMI < 18.5) showing an increase in serum NfL levels in comparison to normal weight individuals [108]. Considering these factors influencing NfL levels, reference intervals, e.g., for different age groups and renal clearance, need to be established to improve NfL interpretation. This is especially important for inter-individual comparison and individual follow-up evaluation in clinical praxis.

## Are we on the track toward clinical implementation of NfL?

Several immunoassays (e.g., Simoa, Ella) are currently available to reliably quantify NfL in blood at very low concentrations [102]. To date, many centers have already implemented the Simoa or a similar platform to measure NfL not only in clinical research, but also to identify neuronal damage in daily clinical routine. Although sample kits are still costly, NfL analysis is already applied in several neurological disorders (e.g., multiple sclerosis, Alzheimer’s disease, amyotrophic lateral sclerosis) for diagnostic or prognostic applications. Here, encouraged by the accumulating evidence for prospective value for disease-associated neuronal damage in various etiologies, neurologists envisage a clear perspective of wider implementation in neurological diseases and beyond. The growing number of platform providers raises hopes that NfL analysis might become more cost-effective in the near future. Acute and individual measurements will become possible due to the increasing and daily use of NfL analysis in a variety of neurological entities and, as NfL is stable in blood samples and can be shipped [109, 110], routine measurements of serum NfL levels are possible in large reference laboratories. Of note, certain limitations exist that are particularly relevant in critical ill patients. These include neuro-axonal injury due to neurological comorbidities or critical illness polyneuropathy, reduced clearance due to renal failure, or interindividual variability (e.g., BMI, blood volume), as detailed above. However, most of these limitations can be addressed by correcting NfL serum levels according to such individual variables and correlation to standard values (see also below).

In this regard, NfL might be valuable by providing information on long-term prognosis, as repeated NfL measurements might help to differentiate between reversible brain dysfunction (e.g., drug-induced delirium) and structured brain damage (e.g., ischemic brain lesions) [56]. Furthermore, NfL might become also highly valuable for evaluating the severity of SAE and direct assessment of neuronal damage in delirium subphenotypes, e.g., hypoactive, hyperactive and mixed delirium [111, 112] and during ambiguous clinical symptoms, such as drug-induced delirium or in sedated patients (e.g., with benzodiazepines, opioids, anticonvulsives) [113]. Considering that hypoactive delirium is associated with worse outcomes as compared to hyperactive or mixed phenotypes, the increase of serum NfL may be helpful in its early diagnosis and prognosis of outcome. At this point, prospective measuring NfL in different delirium subphenotypes is a matter for future studies.

**Fig. 3:**
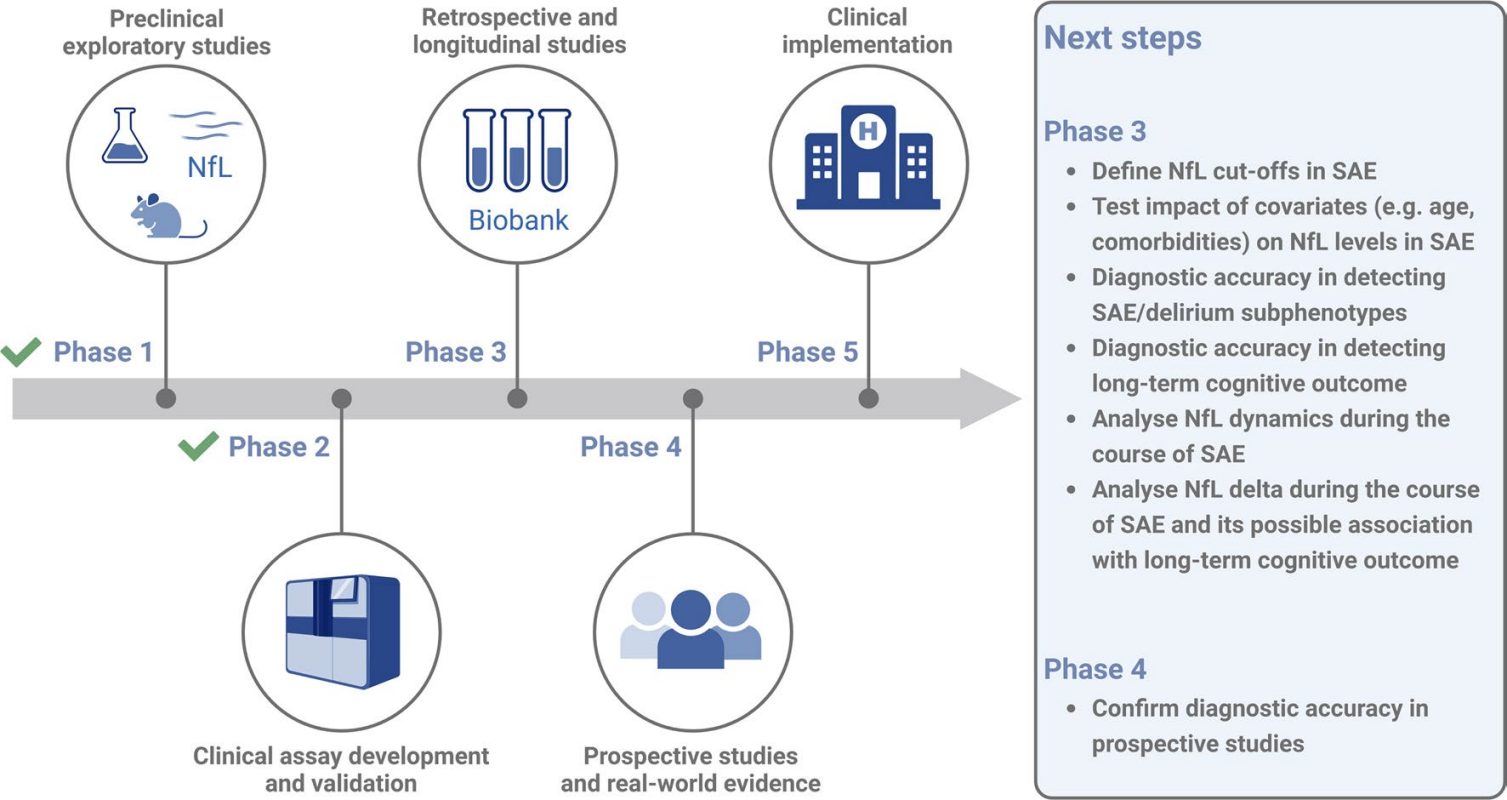
Five phases in the development of blood NfL as a possible biomarker in SAE

In 2001, Pepe and colleagues published a five-phase framework for cancer biomarker development, which has been modified by the Geneva Task Force for the Roadmap of Alzheimer’s Biomarkers for the development of biomarkers [114, 115]. According to these guideline, preclinical exploratory studies and clinical assay development/validation are required in phase 1 and 2. This is followed by retrospective studies using longitudinal data (phase 3), most of which are available in repositories. Prospective studies and real-world evidence will test the biomarker in phase 4, followed by phase 5, which focuses on clinical implementation in daily clinical routine (Fig. 3) [73]. For NfL as a biomarker, phase 1 and 2 have already been accomplished. For the application in SAE, phase 3 is ongoing and additional prospective and well-powered studies in phase 3 and 4 should answer the questions a) if an increase in NfL is associated to long-term neurocognitive outcome in sepsis survivors and b) if increased serum NfL levels are able to differentiate between delirium subphenotypes and predict SAE-related neuronal damage. A successful clinical implementation requires distinct reference limits. The use of age-adjusted reference limits or percentiles and z-scores has already been attempted and partially implemented in the laboratory practice [116, 117]. These reference values need to be confirmed and implemented for general use and standard values for NfL adjusted for comorbidities should be established for regular use in critical ill patients. Future prospective studies with an appropriate sample size should also standardize, include, and compare clinical criteria and assessment tools for SAE with respect to different subphenotypes of delirium for analysis together with NfL serum levels.

## Data Availability

Not applicable.

## Abbreviations

SAE: Sepsis-associated encephalopathy
NfL: Neurofilament light chain
ICU: Intensive care unit
CSF: Cerebrospinal fluid
CAM-ICU: Confusion Assessment Method for the Intensive Care Unit
Nu-DESC: Nursing Delirium Screening Scale
GCS: Glasgow coma scale
ICDSC: Intensive Care Delirium Screening Checklist
RASS: Richmond Agitation-Sedation Scale
DSM: Diagnostic and statistical manual of mental disorders
CRS: Confusion Rating Scale
NSE: Neuron-specific enolase
GFAP: Glial fibrillary acidic protein
UCH-L1: Ubiquitin carboxy-terminal hydrolase-L1
ICU-AW: ICU–acquired weakness
CINM: Critical illness neuromyopathy
BMI: Body mass index
